# Leaflet by Leaflet Synergistic Effects of Antimicrobial Peptides on Bacterial and Mammalian Membrane Models

**DOI:** 10.1021/acs.jpclett.3c00119

**Published:** 2023-04-19

**Authors:** Arpita Roy, Nirod Kumar Sarangi, Surajit Ghosh, Amrutha Prabhakaran, Tia E. Keyes

**Affiliations:** School of Chemical Sciences and National Centre for Sensor Research, Dublin City University, Glasnevin, Dublin 9, Ireland

## Abstract

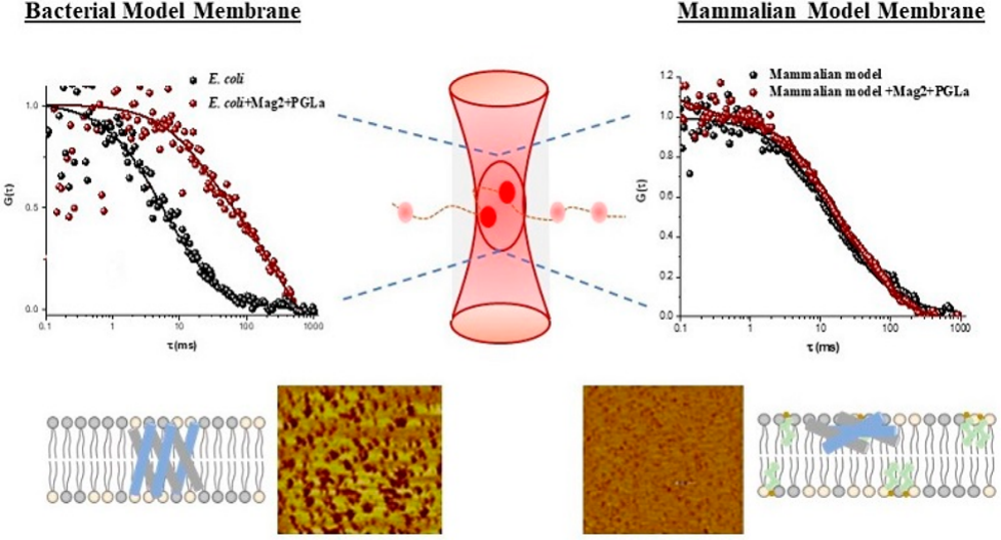

Antimicrobial peptides (AMPs) offer significant hope in the fight against antibiotic resistance. Operating via a mechanism different from that of antibiotics, they target the microbial membrane and ideally should damage it without impacting mammalian cells. Here, the interactions of two AMPs, magainin 2 and PGLa, and their synergistic effects on bacterial and mammalian membrane models were studied using electrochemical impedance spectroscopy, atomic force microscopy (AFM), and fluorescence correlation spectroscopy. Toroidal pore formation was observed by AFM when the two AMPs were combined, while individually AMP effects were confined to the exterior leaflet of the bacterial membrane analogue. Using microcavity-supported lipid bilayers, the diffusivity of each bilayer leaflet could be studied independently, and we observed that combined, the AMPs penetrate both leaflets of the bacterial model but individually each peptide had a limited impact on the proximal leaflet of the bacterial model. The impact of AMPs on a ternary, mammalian mimetic membrane was much weaker.

Antibiotic resistance is a worldwide health concern because it has drastically reduced the therapeutic efficiency of conventional antibiotics.^1,2^ Pathogenic microbes have developed resistance against antibiotics due to their long-term, indiscriminate overprescription in human and animal therapy. This, along with the dramatic decline in the introduction of new antibiotics, has led to a crisis in the treatment of microbial, especially bacterial, disease.^3^ The available therapeutic options in the market for antibiotic-resistant bacterial infections have diminished dangerously, and there is an urgent need to find alternative therapeutics.^4^

Antimicrobial peptides (AMPs) are considered highly promising candidates for the treatment of drug-resistant pathogens and are attractive alternatives to conventional antibiotics due to their broad spectrum of antimicrobial activity.^5−7^ AMPs are small, cationic, amphiphilic α-helix-forming proteins that, for a number of organisms, form a first line of defense and an essential part of their innate immune system. AMPs target the physiochemical properties of negatively charged bacterial membranes, in contrast to the action of most conventional antibiotics, which disturb the metabolic pathways of bacteria. Consequently, pathogenic bacteria are unable to develop resistance against AMPs.^8,9^

PGLa and magainin 2, isolated from the skin of the African clawed frog *Xenopus laevis*, were some of the earliest AMPs to be discovered.^10,11^ Both damage bacterial membranes, but in particular, it has been shown that combined, PGLa and magainin 2 exhibit intriguing synergistic effects attributed to membrane pore formation.^12,13^ Electrostatic interactions between the positively charged protein and the negatively charged membrane surface are responsible for the early binding of the AMP with the target membrane. The antimicrobial activity begins once a threshold concentration is exceeded following the completion of initial binding. Conformational changes, self-association, and/or multimerization occur in the peptides after they engage with bacterial membranes, that can be hard to observe experimentally. A membrane-active peptide’s mechanism of action depends on its biochemical and structural properties and the target membrane’s characteristics (surface charge, lipid composition, membrane fluidity, and curvature of lipids). However, many AMPs are not sufficiently selective and thus are toxic to host mammalian cells, because some membrane properties (polar headgroup charge, fluidity, packing, etc.) and components (lipid composition) are shared by cells across species. Understanding the molecular mode of action of AMPs using bacterial and mammalian model mimetics is therefore useful in designing new membrane-specific molecules, as an AMP candidate of interest should show minimal to no activity against mammalian cells, with maximum activity against bacterial membranes for therapeutic use. Because a key mode of action of AMP is membrane poration, pore formation, and its structure, and size are of particular interest but necessitate nanoscale resolution approaches; thus, biophysical strategies are necessary to understand these processes at this scale.

Cell-based and liposomal biophysical assays have both been used to examine the interaction of AMPs with biomembranes in an effort to deduce the mechanism of antimicrobial activities and to link them with the structural features of peptide–membrane association.^14−18^ Cell-free planar biomembrane platforms are particularly attractive as they can provide predictive, mechanistic insights into membrane–peptide interactions without the complexities of working with live bacteria, and they can be amenable to rapid and high-throughput formats that can be applied to iterative design.

Artificial membranes are frequently used to investigate membrane-associated cellular processes in a controlled way.^19,20^ Biomimicry in such systems requires a delicate balance between experimental approaches and physiological relevance. Substrate-supported lipid bilayers have proven to be indispensable tools for mimicking cellular membranes with a controlled composition and versatile analytical addressability.^21−24^ However, the interaction of a solid support with the membrane lower leaflet or with a reconstituted protein restricts the fluidity of the membrane components, limiting studies involving protein/peptide mobility or leaflet by leaflet interrogation. Such substrate effects may influence AMP’s transverse orientation and mobility in a lipidic environment, which in turn may have an influence on its predicted activity at the target membrane. Microcavity-supported lipid bilayers (MSLBs) provide a good balance between membrane stability, compositional versatility, fluidity, and analytical addressability and can be used without the limitations of frictional substrate interactions to enable leaflet by leaflet interrogation of membrane diffusivity. In this format, the biomimicking lipid bilayer, prepared using Langmuir–Blodgett and vesicle fusion methods, spans buffer-filled micrometer-sized spherical pores, that enable control of the lipid compositions on gold and PDMS substrates and allows monitoring of membrane behaviors via electrochemical^25−27^ and optical readouts.^26^

Herein, we use MSLBs to investigate the interaction of PGLa, magainin 2, and their 1:1 mixture with *Escherichia coli* extract membrane and a ternary lipid membrane (DOPC/SM/cholesterol) that mimic the bacterial and eukaryotic plasma membranes, respectively. Electrodes made of MSLB arrays on gold are used for electrochemical interrogation of membrane behaviors in response to AMP interactions. The concentration-dependent change in the resistance and capacitance of the membrane was systematically monitored after the addition of AMPs individually and as a mixture. PGLa and magainin 2 have been shown to work synergistically with an effect that is more prominent at the biomimetic *E. coli* extract membrane than the mammalian lipid composition. Alterations in membrane structure caused by AMP and their synergistic effect are studied by atomic force microscopy (AFM), and the results correlate strongly with the electrochemical impedance spectroscopy (EIS) measurements.

The flexibility of the MLSB platform allowed us for the first time to investigate how the AMPs individually, and in combination, affect the diffusivity of each bilayer leaflet. This was accomplished by determining the lateral diffusion of labeled lipid markers separately at the inner and outer leaflets of MSLBs on PDMS substrates using fluorescence correlation spectroscopy (FCS). Therefore, this study establishes the different orientation of the individual antimicrobial peptides and their mixture in both bacterial and mammalian model membranes. Moreover, in this Letter, we compare the effects of AMPs synergism on bacterial and mammalian membranes using a multimodal approach and present a molecular interpretation for the observed differences.

First, EIS measurements were performed on a bilayer composed of the *E. coli* extract and a ternary lipid composition [DOPC/SM/Cholesterol (4:4:2 moles)] as mimics of the bacterial and mammalian membranes, respectively. The corresponding relative changes in resistance and capacitance for the mixed AMPs are shown in panels a and b, respectively, of Figure 1. Membrane resistance and capacitance changes were evaluated for individual AMPs at concentrations ranging from 100 nM to saturation (∼2 μM), which corresponds to the range previously reported to cause membrane pore formation.^18,28^ The corresponding data are shown in Figure S1a,b. In a separate experiment, we confirmed that incubation at each concentration for 30 min is sufficient to achieve an electrochemical steady state, as shown in Figure S2.

**Figure 1:**
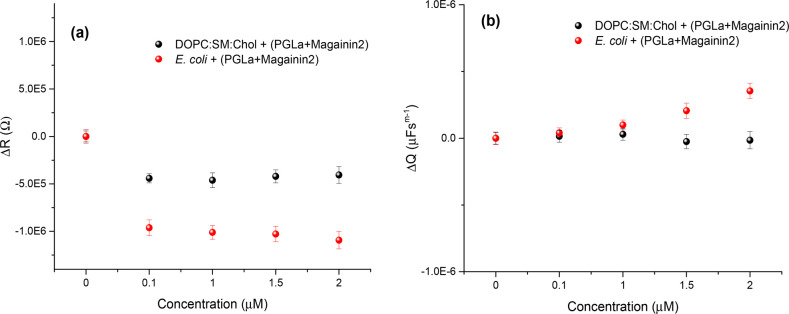
Relative variation of (a) resistivity (Δ*R*) and (b) capacitance (Δ*Q*) of the *E. coli* and mammalian model membrane bilayer upon treatment with an equimolar mixture of magainin 2 and PGLa. All EIS measurements were performed within the frequency range of 0.05–10^5^ Hz at a dc bias voltage of 0 V with an ac amplitude of 0.01 V in 0.01 M PBS at 22 ± 1 °C. The data are means ± SD. The two peptides were premixed at an equimolar ratio in these experiments and then introduced into the electrochemical cell containing the MSLB systems. After incubation at each concentration for 30 min, data points were recorded.

The EIS response indicates that the *E. coli* bilayer resistance decreases upon incubation with the individual AMPs, magainin 2 or PGLa, consistent with increased membrane admittance. This reduction in bilayer resistance is consistent with a previous EIS study of magainin I.^29^ It is noteworthy that the extent of the decrease in resistance for PGLa is double that of magainin 2 (Figure S1a), indicating that PGLa and magainin 2 interact differently with the MSLB, due to differences in the binding affinity and/or orientation of the peptide within the membrane. We speculate that this is because magainin 2 partitions less deeply into the membrane and remains at the MSLB surface, whereas PGLa penetrates more deeply into the MSLB. This is consistent with a report in which the smaller hydrophobic sector of magainin 2 compared to that of PGLa prevents magainin 2 from inserting as deeply into the bilayer.^30^ Moreover, it is consistent with reported variation in how the AMPs are oriented at the membrane, which will be discussed in detail below.^12,30,31^ Notably, the bilayer electrochemical resistance is dramatically decreased (Δ*R* is >3-fold lower than that with only magainin 2) when PGLa and magainin 2 are applied as an equimolar mix to the bilayer (Figure 1a). This can be attributed to the synergistic interaction of the combined peptides with the bilayer and is suggestive of pore formation. From nuclear magnetic resonance (NMR) and other studies, it is hypothesized that combined, the AMPs form heterodimers that traverse the membrane in the inserted I state^31^ and may further assemble into a stable toroidal pore, facilitating the passage of ions through the bilayer pores.

The trend in changes to bilayer capacitance is also notably different for each peptide and their mixture (Figure S1b). The *E. coli* bilayer after incubation with magainin 2 showed the largest increase in capacitance (Δ*Q* = 0.4 × 10^–6^ Fs^m-1^ at 1 μM magainin 2). Given the reciprocal relationship between membrane thickness and capacitance,^32^ it is reasonable to conclude that magainin 2 causes membrane thinning due to changes in the acyl chain layer thickness or modifications to the bilayer area due to the preferred orientation of magainin 2 at the bilayer interface.^30^ This observation is consistent with literature reports indicating magainin 2 follows the carpet model, with the peptide aligned parallel to the bilayer surface.^33^Figure S1b shows that PGLa alone induces an only marginal change in capacitance (Δ*Q* = −0.08 × 10^–6^ Fs^m-1^ at 1 μM). The decrease, though within experimental error, suggests the PGLa causes some membrane thickening. In contrast, when the two peptides are administered together, Figure 1a,b, there is a dramatic impact on membrane resistance and capacitance, indicating a rearrangement of the peptide orientation and much deeper penetration into the bilayer. This can be attributed to the formation of a heterodimer between magainin 2 and PGLa, which are hypothesized to assemble and penetrate into the bilayer spanning both membrane leaflets.^31^ This assembly has been shown to lead to transmembrane leakage attributed to the formation of the toroidal pore,^12,34^ which is consistent with the large decrease in *E. coli* bilayer resistance and the increase in capacitance observed here.

Given the membrane interaction of the AMPs and the synergism evident at the *E. coli* membrane, we were curious to compare the impact at a mammalian membrane analogue. Interestingly, the overall decreases in resistance induced by the combined AMPs at mammalian MSLBs are much smaller than those of the bacterial analogue. The mixture of the two peptides elicited a decrease in membrane resistance that was 2-fold greater for bacterial than for mammalian analogues: *E. coli* membrane (Δ*R* ∼ 1.09 MΩ) compared to the mammalian system (Δ*R* ∼ 0.406 MΩ). This can be attributed to the negatively charged phospholipids in the *E. coli* composition that facilitate electrostatic binding with the cationic AMPs. Such effects are absent in the mammalian plasma membrane in the absence of charged lipids.^35,36^ In addition, the rigidifying effect of cholesterol on the mammalian composition also likely offers protection to the membrane against the admittance changes induced by AMPs.^37^ The capacitance of the mammalian MSLB remains essentially constant (Figure 1b), indicating that, despite the modest decrease in resistance, any alteration in membrane organization is not accompanied by changes in membrane thickness.

To better comprehend the distinction between carpet-like assemblies and toroidal pore formation by individual and combined AMPs, AFM studies were performed. Widely used for biophysical models, it offers a powerful approach for imaging changes in membrane structure at nanoscale resolution.^38−46^ We examined the impact of individual 2 μM magainin 2 and 2 μM PGLa, as well as the peptide mixture, at a total concentration of 2 μM (1 μM each) at an *E. coli* bilayer supported on mica; the concentration was selected to enable direct comparison with the results of the EIS study. Panel a of Figure 2 shows a representative topographic image of the *E. coli* bilayer surface before AMP addition, and panels b and c of Figure 2 show the corresponding images upon incubation with magainin 2 and PGLa, respectively. Without AMP, the *E. coli* membrane surface shows modest lateral heterogeneity with submicroscopic domains protruding at a height of ∼1.4 nm attributed to the cardiolipin lipid (Figure 2a,e).

**Figure 2:**
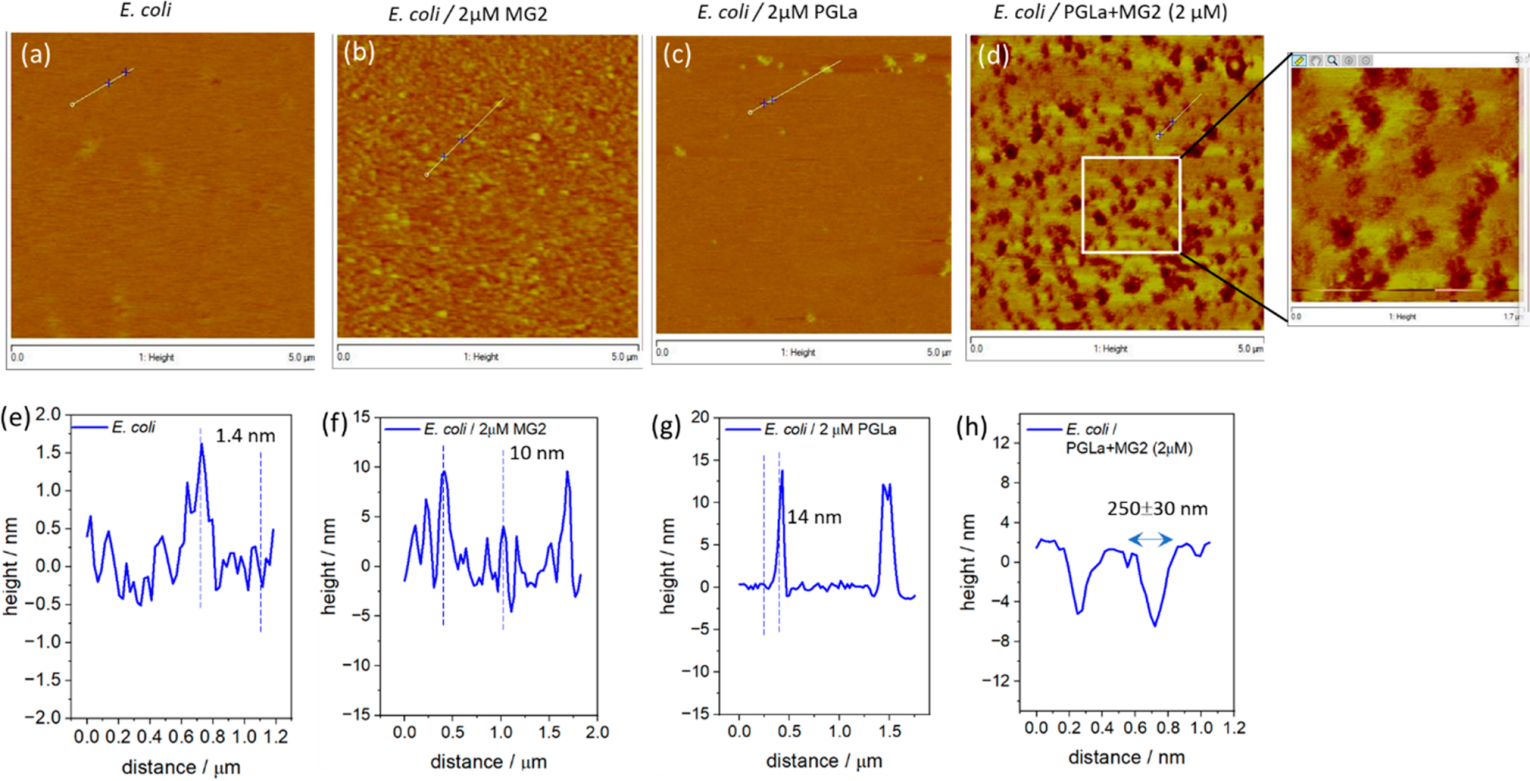
Representative AFM images of the *E. coli* bilayer (a) without AMPs or in the presence of (b) 2 μM magainin 2 (MG2), (c) 2 μM PGLa, and (d) an equimolar mixture of magainin 2 and PGLa (1 μM maganin 2 and 1 μM PGLa) with a total concentration of 2 μM in the contact buffer. The line scans across images a–d are shown in panels e–h, respectively. Images b–d were captured following peptide incubation for 30 min.

From the AFM images (panels b and c of Figure 2) and the corresponding line scans shown in panels f and g, respectively, of Figure 2, there is no evidence for the formation of transmembrane pores upon incubation with individual peptide for 30 min, at the concentrations explored here, even at the highest concentration of 2 μM. Nonetheless, it is clear that the individual peptides loosely assemble at the bilayer surface consistent with a carpet-like mechanism. This is consistent with literature reports on magainin 2–membrane interactions in this lower-concentration range that also indicate that magainin 2 primarily binds at the membrane surface and embeds only shallowly.^30^ Membrane pore formation has been noted to be induced by magainin 2, which was observed at a much higher peptide concentration (400 μM).^47^ In contrast, PGLa forms oligomeric clusters seen as protrusions with a height of ∼14 nm (Figure 2g), attributed to their assembly at anionic cardiolipin domains, but there is no evidence of large-scale pore formation, which is consistent with our EIS observations. It is noteworthy that while we can observe PGLa aggregates only by AFM, most of the PGLa is expected to be associating homogeneously at the bilayer (Figure 2c). Even after prolonged incubation, neither peptide formed pores; however, when the image was captured after binding of PGLa to the *E. coli* membrane for 1 h, the PGLa oligomers remained partially embedded, potentially residing in a tilted (T) conformation within the bilayer, as reflected by a decrease in oligomer height from 14 to 3 nm in the line profile scan (Figure S3a).

In dramatic contrast, as shown in Figure 2d, transmembrane pores are very clearly evident when magainin 2 and PGLa are incubated together at the membrane. This is direct evidence of the distinctive “I” conformation that originated from to synergistic effect that AMPs have, when combined, on the bacterial membrane composition. From Figure 2h, a line scan across the image in Figure 2d, provides an approximation of the average pore diameter of ∼250 ± 30 nm (20–25 pores analyzed; *N* = 3), which is likely due to pore–pore coalescence. Indeed, we observed that pore formation in the *E. coli* membrane is triggered by a synergistic impact that begins as soon as 5 min into the incubation (Figure S3b). Although the pore dimensions are <100 nm at the start of the incubation, they grow larger over time, perhaps as a result of pore–pore coalescence. Such large-scale toroidal pore formation, as reported previously, is typically initiated by the formation of heterodimers of both AMPs that act as nucleation sites leading to larger-scale structures.^31^ Our observations correspond well with those from an electron microscopic study on *E. coli* cells with a different AMP, in which the formation of large pores with comparable dimensions (∼300 nm) was reported.^48^ Furthermore, it is noteworthy that the synergism from mixed peptides is observed at a 50% lower concentration of 1 μM (0.5 μM magainin 2 and 0.5 μM PGLa), with evidence of toroidal pore formation (Figure S3c), albeit with a pore dimension (<100 nm) relatively smaller than that of combnied concentration of 2 μM.

The effect of the mixture of magainin 2 and PGLa was then compared at the neutral, cholesterol-containing mammalian model membrane. As expected, the ternary membrane (DOPC/SM/Chol) displays the liquid-ordered (L_o_), seen as brighter patches and liquid-disordered (L_d_) phase domains before incubation with the peptide mixture (Figure 3a). Notably, treatment with the mixed peptide homogenizes the membrane and shows evidence of pore formation. This suggests that the effects of the combined AMPs are not localized to the site of action and are insensitive to phase. Instead they disrupt the overall heterogeneity of the lipid bilayer by restructuring adjacent phases, much like pore-forming toxins from bacteria.^21,49^ However, the pores formed in DOPC/SM/Chol are much smaller (∼150 ± 20 nm) (Figure 3b,c) than those at the *E. coli* membrane. The pore depth in bacterial membranes was determined to be 6–7 nm (Figure 2h), but only 1–3 nm (Figure 3c) at the mammalian membrane. This indicates that pore formation occurs through both leaflets of the bacterial model bilayer but is limited to the outer leaflet of the mammalian membrane.^50^ Again, this is consistent with our EIS data that showed significantly smaller changes in resistance and capacitance at mammalian over *E. coli* membrane models.

**Figure 3:**
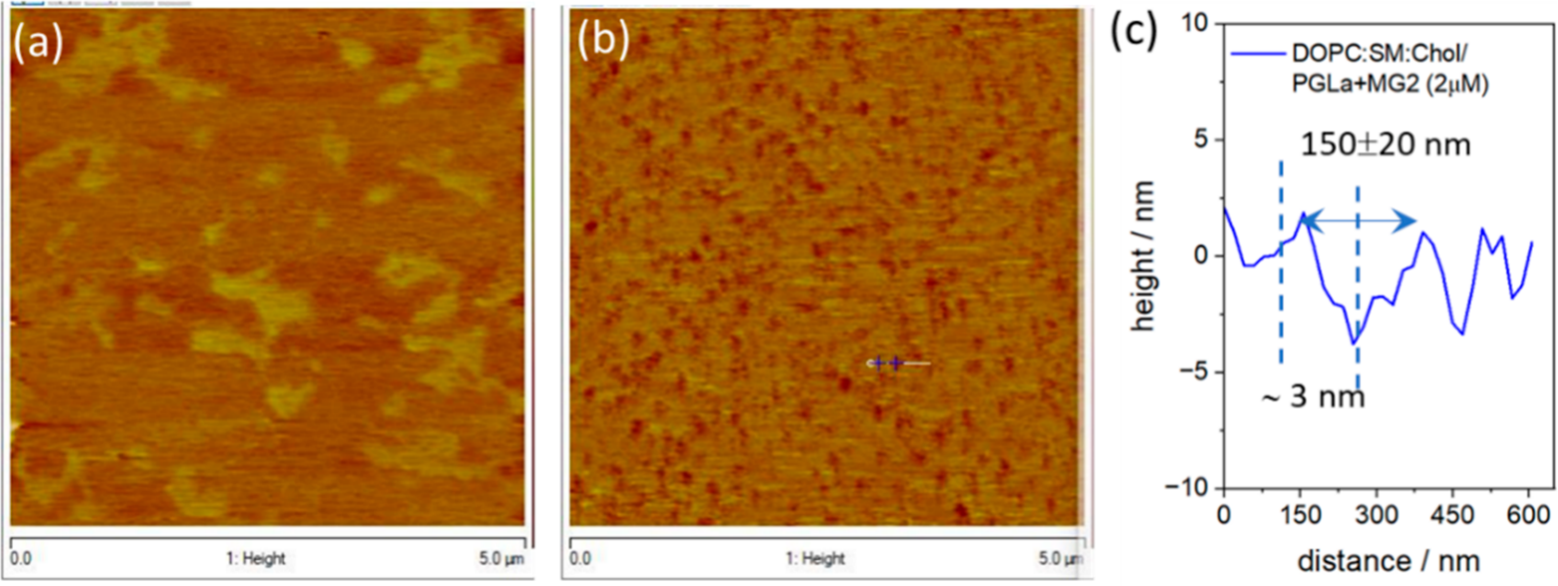
AFM images of a ternary DOPC/SM/Chol (2:2:1) bilayer (a) in the absence and (b) in the presence of an equimolar mixture of magainin 2 and PGLa with total concentration of 2 μM. (c) Line scan across image b.

To investigate further the depth of penetration of the AMPs across the bilayer, we used FCS to monitor how AMP affects lateral diffusion of the lower and upper leaflet at each membrane model. FCS enables direct measurement of lateral lipid diffusion.^28,51−53^ The unique fluidity and layer by layer construction of the membrane in the MSLB platform, enables facile study of each individual leaflet’s diffusivity when the leaflets are doped with distinct fluorophores. FCS was previously used in specific leaflet labeling to study the interaction of pore-forming toxin and leaflet-specific dynamics.^54^

The lateral diffusion of the *E. coli* bilayer membrane in the presence and absence of PGLa and magainin 2 and following incubation with an equimolar mixture of two AMPs at 2 μM was measured in membranes in which ATTO655-DOPE and ATTO532-DOPE labeled the upper and inner bilayer leaflets, respectively. The experiments were carried out at 2 μM AMP, which corresponds to the highest concentration used in AFM and EIS studies. The results of experiments at lower AMP concentrations are shown in Figure S4. From the ACFs, Figure 4a, and Table S1, it is clear that the rate of diffusion of the upper membrane leaflet is reduced in the presence of both individual peptides. The diffusion coefficient (*D*) of the pristine *E. coli* bilayer, for example, is ∼7.9 ± 0.60 μm^2^ s^–1^ compared to 6.1 ± 0.51 μm^2^ s^–1^ in the presence of 2 μM magainin 2. Such modestly reduced diffusivity is consistent with magainin 2’s surface assembly in the S state at the bilayer (as shown schematically in Figure 5a)^31^ through electrostatic interaction with the negatively charged headgroup of the *E. coli* lipids. In the presence of PGLa, the rate of lateral diffusion of the upper leaflet is dramatically reduced. We fitted the ACF curve with a two-component two-dimensional Brownian diffusion model, and the corresponding diffusivity values from the faster and slower components are 2.3 ± 0.43 μm^2^ s^–1^ (90%) and 0.1 ± 0.03 μm^2^ s^–1^ (10%), respectively. When compared to that with magainin 2, the PGLa-induced reduction is greater, implying a deeper penetration of PGLa, likely residing in a tilted T-shaped orientation,^31^ into the bilayer (Figure 5a), which is consistent with a previous report.^31^ Moreover, the dramatic decrease in the highest-amplitude diffusion coefficient in the case of PGLa is likely attributed to this AMP’s deeper penetration into the membrane compared to magainin 2. The very slow component may be due to the influence of aggregated PGLa. Crucially, the *D* for the lower (proximal membrane) leaflet, as measured by DOPE-ATTO532 diffusivity, is much higher (∼5.9 ± 0.80 μm^2^ s^–1^) and remains constant within the experimental time frame of membrane incubation with the individual AMPs (Figure 4b).

**Figure 4:**
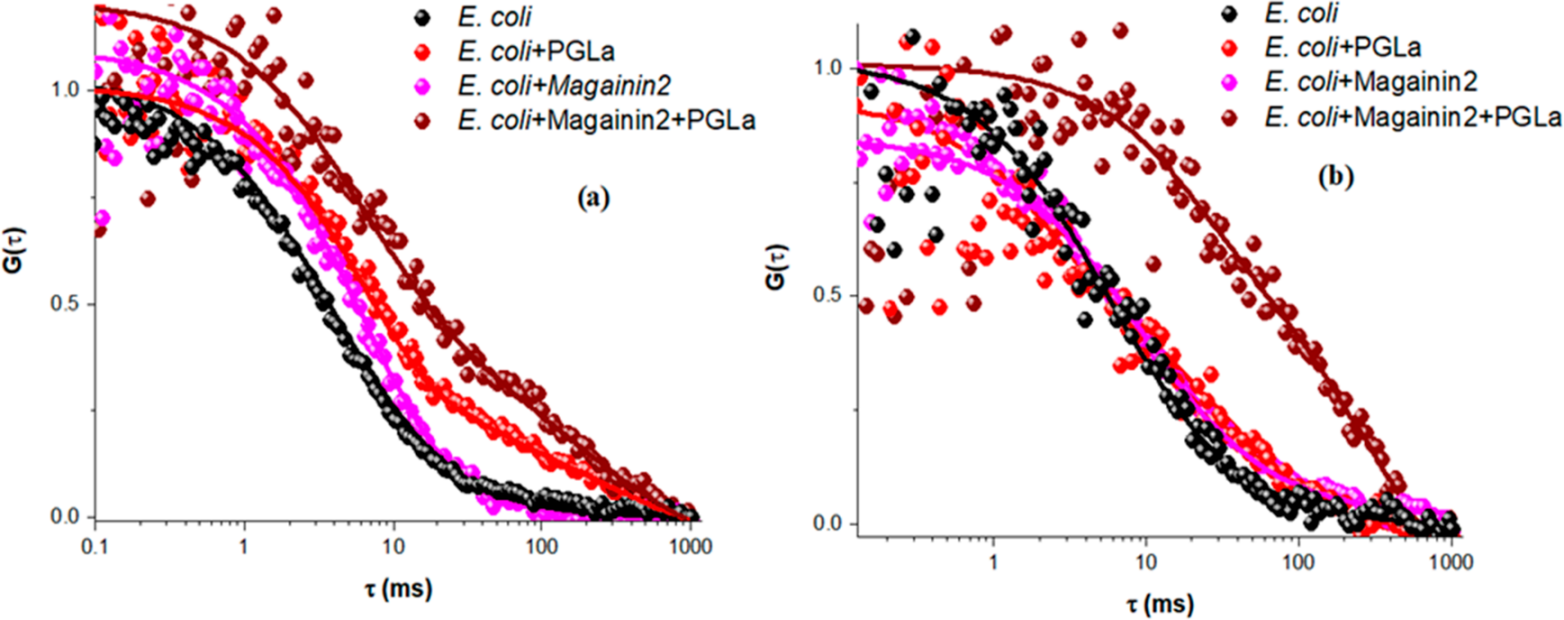
Representative autocorrelation functions (ACFs) obtained for labeled (a) DOPE-ATTO655 (in the upper leaflet) and (b) DOPE-ATTO532 (in the lower leaflet) *E. coli* MSLBs in the absence of AMPs (black circles) or in the presence of PGLa (red circles), magainin 2 (pink circles), and an equimolar mixture of magainin 2 and PGLa (brown circles) with a concentration of 2 μM in each case.

**Figure 5:**
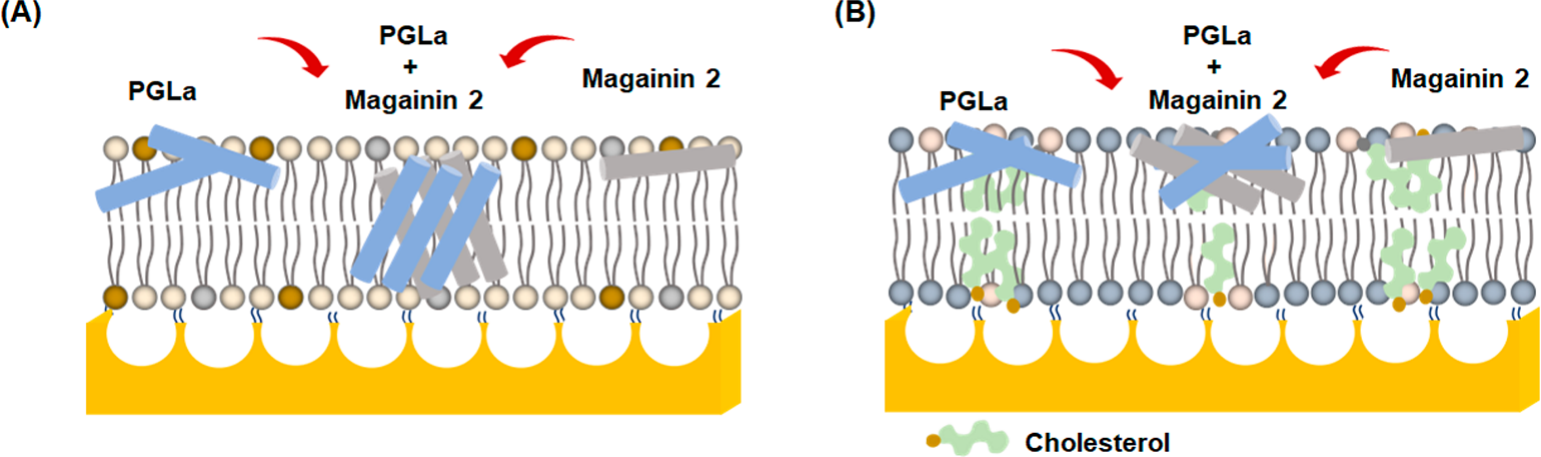
Schematic representation of a microcavity-supported lipid bilayer (MSLB) array and plausible membrane–peptide association in model bilayers of (A) a bacterial membrane (*E. coli* extract) and (B) a mammalian membrane (DOPC/SM/cholesterol).

Again, consistent with EIS and AFM data, the impact of AMP of membrane diffusivity is most significant when the peptides are combined. Upon incubation with the AMP mix, the diffusivity of upper leaflets was dramatically impeded [D of 1.84 ± 0.54 (80%) and 0.1 ± 0.02 (20%)], which is consistent with the hypothesized change of T-shaped orientation of PGLa to an I-shaped orientation (Figure 5a) in the presence of an equimolar quantity of magainin 2. This transformation from the T- to I-state orientation agrees very well with literature reports on PGLa/magainin 2 mixtures.^31^ The lateral diffusion of the lower leaflet, largely unaffected by individual AMPs, now decreases by over an order of magnitude to 0.56 ± 0.26 μm^2^ s^–1^ (Table S1). The anomalous coefficient, α, remained at approximately 1 across all studies, indicating Brownian motion. This demonstrates unequivocally that a transmembrane pore involving both inner and outer leaflets is formed at this low micromolar concentration of AMP only when PGLa and magainin 2 act synergistically.

The impeded diffusivity observed during the FCS study is consistent with an earlier NMR study by Wi et al., who found that the rate of lateral diffusion of lipids decreased in the presence of aurein-3.3 and magainin 2.^34^ The authors hypothesized that the presence of membrane-acting peptides impedes the rate of lateral lipid diffusion in membranes due to electrostatic, hydrophilic, and/or hydrophobic peptide–lipid interactions.^34^ They stated that membrane surfaces bound with AMPs would have significantly lower rates of lateral diffusion. However, it was less obvious why lipids involved in the holes of toroidal pores move more slowly (<10^–11^ cm^2^/s) than lipids involved in thinning membrane bilayers (10^–8^ to 10^–9^ cm^2^/s).^34^ Their hypothesis also states that the lateral diffusive motion of lipids in a toroidal pore would be significantly slower than that of pure bilayers because the lipid motion would have to accompany somewhat unfavorable transbilayer motions involving both up and down leaflets, while those occurring on a thinned membrane surface would still maintain a reasonably fast rate of diffusion because the mechanism involved is the typical lateral diffusive motion of lipids on a single surface. This reasoning is also supported by the observation by Opella and co-workers that membrane proteins incorporated in bicelles undergo fast axial diffusion (*D*_rot_ ≥ 10^5^ s^–1^) on the bicelle surface, providing narrow line widths along the ^15^N chemical shift (<2 ppm) and ^1^H–^15^N dipolar couplings (∼250 Hz).^55^

To the best of our knowledge, there has been no direct exploration of the influence of AMPs selectively at membrane outer and inner bilayer leaflets. Such capacity to interrogate each leaflet independently in a highly fluidic bilayer system is a key advantage of the MSLB platform.

In the case of mammalian MSLB (Table S2), as expected, the diffusivity of this ternary mixture in the absence of AMP is considerably lower than that of the *E. coli* membrane at 3.7 ± 0.58 μm^2^ s^–1.^ The rate of lateral diffusion of the upper leaflet (ATTO655-DOPE) decreases modestly upon incubation with magainin 2 to 3.3 ± 0.41 μm^2^ s^–1^ and upon incubation with PGLa to 2.8 ± 0.37 μm^2^ s^–1^. It slows further for the mixture of peptides (Figure S5a). However, the effects are confined to the distal leaflet for the mammalian membrane (Table S2). Figure S5b shows that the ACFs for the lower leaflet did not change upon incubation with AMP for the mammalian membrane, even in the presence of the peptide mixture.

This is taken to indicate that transmembrane pore formation involving both inner and upper leaflets is not occurring at the mammalian MSLB (Figure 5b). This is highly consistent with AFM imaging that also showed that even together, the peptides do not penetrate deeply into the mammalian bilayer.

Finally to gain some structural insight into the association of the peptides with the membrane interface, we examined the association of the peptide with the membrane interface by surface-enhanced Raman spectroscopy. The microcavity arrays on gold substrates are excellent SERS substrates.^27,56^ We used Raman spectroscopy here to probe the synergic effects of mixed antimicrobial peptides on an *E. coli* extract membrane. Classical Raman spectra of the *E. coli* extract (mixture of POPG, POPE, and cardiolipin) were recorded for powder samples on flat gold substrates (Figure S6a). The observed Raman peaks are characteristic of phospholipids and in good agreement with previous reports.^56−58^ The Raman peak positions of the powder *E. coli* extract are assigned to compare with the band positions with the *E. coli* bilayer and in the presence of mixed peptides. The SERS spectra of the *E. coli* bilayer and *E. coli* bilayer with mixed peptides are shown in panels b and c, respectively, of Figure S6. The presence of peptides in the *E. coli* bilayer is indicated from SERS spectra with the appearance of intense bands in the range of 1000–1200 and 1655 cm^–1^, related to phenylalanine and amide I peaks along with the lipid peaks. We also performed similar Raman experiments with the ternary bilayer. For the sake of comparison, the Raman signals for powdered lipids are also provided (Figure S7a–c). However, the Raman peak intensities of the ternary bilayer remain almost unchanged in the presence and absence of mixed peptides (Figure S7d,e). This is consistent with FCS and AFM measurements and indicates that the mixed peptides interact strongly with the upper and lower leaflet of the *E. coli* bilayer, where they penetrate sufficiently deeply to access the metal plasmonic field, resulting in their SERS enhancement. Conversely, at the mammalian membrane the peptide assembly remains confined to the outer leaflet and therefore does not penetrate deeply enough to undergo SERS enhancement.

In summary, multimodal interrogation of the molecular mechanism behind the synergism between interaction of AMPs, PGLa, and magainin 2 was carried out at microcavity- and mica-supported bilayers. EIS and AFM data showed that when combined, PGLa and magainin 2 induced dramatically greater changes in resistance and capacitance at both membrane types, compared to the individual peptides. The synergistic effect was found to be due to pore formation occurring when the AMPs act in concert, and the effect was strongest at *E. coli* membranes. This was attributed to the observation from AFM depth profiling that the pore penetrates the outer leaflet only the mammalian composition but spans the bilayer in *E. coli* composition. This conclusion was supported by an FCS study in which leaflet by leaflet diffusion of the membranes were studied at the MSLB to examine the impact of the individual and combined peptides. The impact and synergism were greatest at the *E. coli* membrane composition where individually the AMPs decreased the diffusivity of the outer, contacting membrane layer, but significant diffusivity changes to the lower leaflet were observed only in the synergistic mixture. Conversely, the impact of the combined AMPs on the membrane diffusion was confined to the outer leaflet only in the mammalian bilayer. The selectivity of the action of AMPs and their synergism toward a bacterial membrane over a healthy mammalian membrane can be explained by the negatively charged surface of bacteria that predominantly consists of phosphoethanolamine, phosphatidylglycerol, and cardiolipin, while mammalian membranes are protected from membrane lysis by these peptides due to the abundance of neutrally charged lipids phosphocholine and cholesterol. Overall, the data are highly consistent with the formation of the hypothesized I state due to self-assembly of the two AMPs at the bacterial membrane. The platform approach discussed here is potentially amenable to high-throughput study and may form a useful route to divining new AMP synergisms in the future.

## Supporting Information Available

Experimental methods for microcavity and MSLB fabrication and additional EIS, AFM, Raman, and FCS data (PDF)
