# Supplementary material for: Leaflet by Leaflet Synergistic Effects of Antimicrobial Peptides on Bacterial and Mammalian Membrane Models
Source: J Phys Chem Lett. 2023 Apr 19;14(16):3920–8. doi: 10.1021/acs.jpclett.3c00119 (PMC10150393; doi:10.1021/acs.jpclett.3c00119)
Supplement: Supplementary file 1 — jz3c00119_si_001.pdf [file jz3c00119_si_001.pdf]

Supporting Information for

The Leaflet by Leaflet Synergistic Effects of Antimicrobial Peptides on Bacterial and Mammalian Membrane Models

Arpita Roy[1], Nirod Kumar Sarangi[†], Surajit Ghosh[†,2], Amrutha Prabhakaran, and Tia E. Keyes*

School of Chemical Sciences and National Centre for Sensor Research, Dublin City University, Glasnevin, Dublin 9, Ireland.

[†]Equally contributed

Corresponding author's email: tia.keyes@dcu.ie

1. EXPERIMENTAL SECTION

1.1. Materials

High purity (>99%) 1,2-Dioleoyl-*sn*-glycero-3-phosphocholine (DOPC), porcine brain N-(octadecanoyl)-sphing-4-enine-1-phosphocholine (SM), cholesterol, *E. coli* extract purchased from Avanti Polar Lipids (Alabaster, Alabama) and used as received. 1,2-Dioleoyl-*sn*-glycero-3-phosphoethanolamine-labeled ATTO655 (DOPEA655), 1,2-Dioleoyl-*sn*-glycero-3-phosphoethanolamine-labeled ATTO532 (DOPEA532) were brought from ATTO-Tec GmbH. Phosphate-buffered saline (PBS) tablets, Magainin 2, and PGLa were purchased from Sigma-Aldrich (Wicklow, Ireland). Aqueous solutions were prepared using Milli-Q water (Millipore, Bedford, MA). Polydimethylsiloxane silicon (PDMS) elastomer was purchased from Dow Corning (Wiesbaden, Germany) and mixed following the supplier's instructions. Silicon wafers coated with a 100 nm layer of gold on a 50 Å layer of titanium were obtained from AMS Biotechnology. The monodisperse polystyrene latex sphere with a diameter of 1 μm was obtained from Bangs Laboratories. The commercial cyanide-free gold-plating solution (TG-25 RTU) was obtained from Technic. All other HPLC-grade reagents were obtained from Sigma-Aldrich and used as received.

1.2. Fabrication of PDMS and Gold Microcavity Arrays

Microfluidic microcavity arrays were constructed in PDMS for the FCS experiments or in gold on silicon wafer for EIS studies by polystyrene sphere templating as per earlier reports.[1,2] In brief, for

[1] Current address: Department Of Biochemistry And Bioinformatics, Gandhi Institute of Technology and Management, Visakhapatnam-530045, Andhra Pradesh, India.
[2] Current address: Department of Chemistry, Gandhi Institute of Technology and Management, Visakhapatnam-530045, Andhra Pradesh, India

the fabrication of gold arrays, the PS microsphere of 1 μm diameter were drop cast into the gold-coated silicon wafers by utilizing the gravity assisted technique to form a highly ordered array. Then, electrochemical deposition was used to deposit gold onto the electrode around the array to 50% of the sphere height.[3,4] Finally, the top interpore surface of the gold array electrodes were selectively modified with self-assembled monolayer (SAM) of 1 mM 6-mercapto-1-hexanol (MH). This SAM is required to promote bilayer stability. Finally, the PS sphere template was removed by washing with THF and ethanol to yield top surface SAM modified gold arrays. The electrodes are sonicated in buffer and kept in contact with working buffer until use.

To fabricate substrates for microscopy, optically transparent PDMS microcavity arrays were prepared by drop casting 4.6 μm PS microsphere onto a mica sheet. PDMS was poured across mica sheet and curing was completed at 90°C for approximately 1 h until the PDMS becomes hard. The resulting PDMS substrate was peeled off the mica and the resulting microcavity array was sonicated in tetrahydrofuran (THF) for about 15 m to remove the PS sphere templates. Prior to filling the cavities with PBS buffer of pH 7.4, the substrate was treated by oxygen plasma for 5 m to render it hydrophilic and then sonicated in PBS buffer for 15 m.

For both gold and polymer arrays the same bilayer deposition procedure was applied; lipid monolayer transfer was accomplished using Langmuir-Blodgett deposition. Usually, 50 μL of lipids (1 mg/mL in chloroform) was added dropwise onto the subphase (Milli Q water, 18.2 MΩ.cm) at room temperature (20 ± 1°C), and chloroform was then evaporated for 7 m. The monolayers were subjected 4 cycles of compression/decompression at a barrier speed of 20 mm/min not exceeding the final surface pressure (Π) of 35 mN m^{-1}. Then, the monolayer is compressed up to 32 mN m^{-1} and held for at least 300 seconds before transfer. Lipid monolayers were transferred onto the hydrophilic gold and PDMS substrates vertically up at a speed of 5 and 10 mm/m respectively. For the FCS study, we labelled the lower leaflet as well by DOPE-ATTO532 which is doped into the lipid compositions and injected during the Langmuir-Blodget technique by which monolayer has been formed. Then, the monolayer formed substrates were immersed in a 0.25 mg/ml liposome solution, and the fusion was allowed to occur for 1 to 1.5 h to form bilayers. The substrate was maintained in contact with liposome solution throughout the fusion. The substrate was washed gently with working buffer (PBS pH 7.4) to remove any unfused liposomes and was kept in contact with this buffer while being transferred to the electrochemical chamber. Similarly, for the PDMS substrate, the chamber was washed 3 to 4 times with working

buffer (PBS pH 7.4) and was kept in contact with this solution until the experiment was completed to prevent the bilayer drying.

1.3. Liposome preparation

Briefly, in this work, liposome fusion was used to form the distal lipid leaflet of microcavity supported lipid bilayers (MSLBs). To prepare the liposomes, stock solutions of all liposome components such as DOPC, brain sphingomyelin and cholesterol 10 mg/ml each, and *E. coli* extract (1 mg/ml) were prepared in chloroform and stored in sealed glass vials at -20°C. Fluorescence labelled DOPE-ATTO655, DOPE-ATTO532 was mixed in a ratio of 50000:1 mol/mol with unlabelled lipids for fluorescence correlation spectroscopy (FCS) studies. For electrochemical measurements, as electrochemical impedance spectroscopy (EIS) is label-free, fluorescent probe was not included during the preparation of liposomes. Aliquots of the appropriate amounts of the stock solutions were mixed in clean amber glass vials and dried under a gentle stream of nitrogen and placed under vacuum for 1 h. The lipids were rehydrated in 1 ml of PBS buffer (pH 7.4) and vortexed vigorously for at least 60 s. Small unilamellar liposomes were prepared by extruding the multilamellar liposome suspension 11x against a polycarbonate membrane (0.1 μm pore size) using a mini hand-extruder (Avanti Polar Lipids). The small unilamellar liposomes were diluted to 0.25 mg/ml. Liposomes composed of SM were extruded at 45°C, above the SM transition temperature, to guarantee that liposomes are in the fluidic state.

1.4. Electrochemical impedance spectroscopy

The electrochemical measurements were performed with a CH760A potentiostat (CH Instruments, USA). A standard 3-electrode cell comprised of gold microcavity suspended bilayer as a working electrode, an Ag/AgCl (1 M KCl) reference electrode and a platinum wire auxiliary electrode. However, herein probe free method is used to avoid interactions with drug in main EIS data.

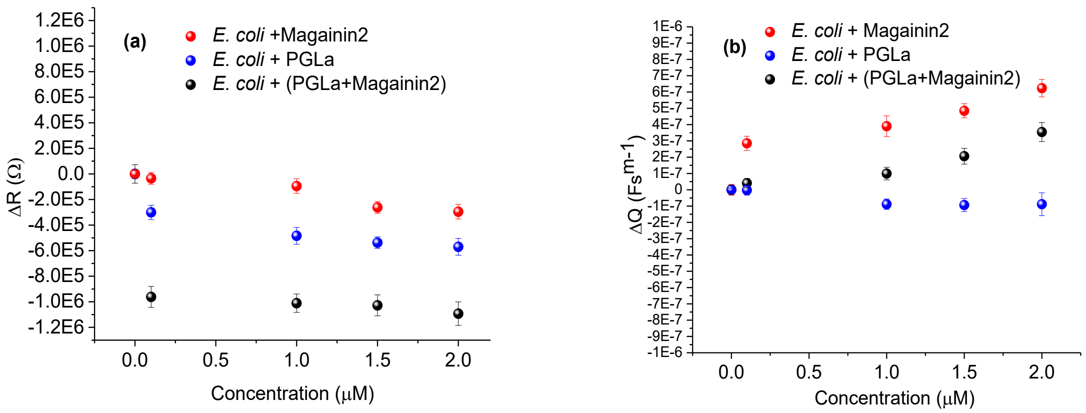

Figure S1: Relative variation of (a) resistivity (ΔR) and (b) capacitance (ΔQ) of the bacterial model membrane bilayer (*E. coli*) upon treatment with Magainin 2, PGLa and the equimolar mixture of PGLa and Magainin 2 (total concentration 2 mM; 1 mM each).

The EIS data were measured over a frequency range of 0.05 to 10^5 Hz with an AC modulation amplitude of 0.01 V at a potential DC bias of 0 V (vs. Ag/AgCl (1 M KCl)). The DC bias of 0 V was selected for all our EIS measurements in 0.01 M PBS, as there is no electrochemical reaction involved. All measurements were carried out in a glass cell (approximate volume of 4 ml) in contact with PBS buffer maintained at pH 7.4. EIS fitting was done by employing Z view (Scribner Associates, v3.4e) software. Typical absolute resistance and capacitance of ternary compositions (DOPC: SM: Chol) forming MSLB prior to peptide incubation were found to be 4.53 ± 0.5 MΩ and 3.8 ± 0.5 μFs^{m-1}respectively.[5-7] Whereas the absolute resistance and capacitance of *E. coli* over MSLB were measured as 2.95 ± 0.3 MΩ and 4.45 ± 0.4 μFs^{m-1}respectively.

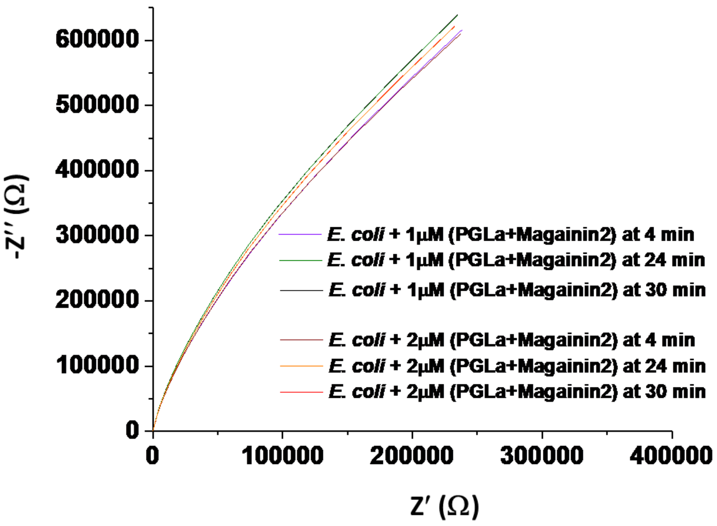

Figure S2: Nyquist plot of *E. coli* membrane with 1 µM and 2 µM mixed peptides in different time intervals, showing steady state is reached for each concentration.

1.5. Atomic Force Microscopy:

Lipid bilayers were imaged using atomic force microscopy (AFM) with a Veeco Bioscope II system coupled with Zeiss Axiovert inverted optical microscope IX70 from Nanotech House in Cambridge, England. Using silicon nitride cantilevers PNP-TR-20 (NANO WORLD) with a nominal force constant of 0.32 N/m and a tip radius of 20 nm, topographic AFM pictures were acquired in PBS buffer in tapping mode. The scan rate was 0.5 Hz while the resonance frequency in the buffer was kept as low as possible during the imaging process. Lipid bilayers comprised of *E. coli* total extracts and DOPC: SM: Chol were prepared on freshly cleaved mica using LB-VF method. Nanoscope 7.30 was used to process the AFM pictures, and a 1st order polynomial was used to plane fit the entire image.

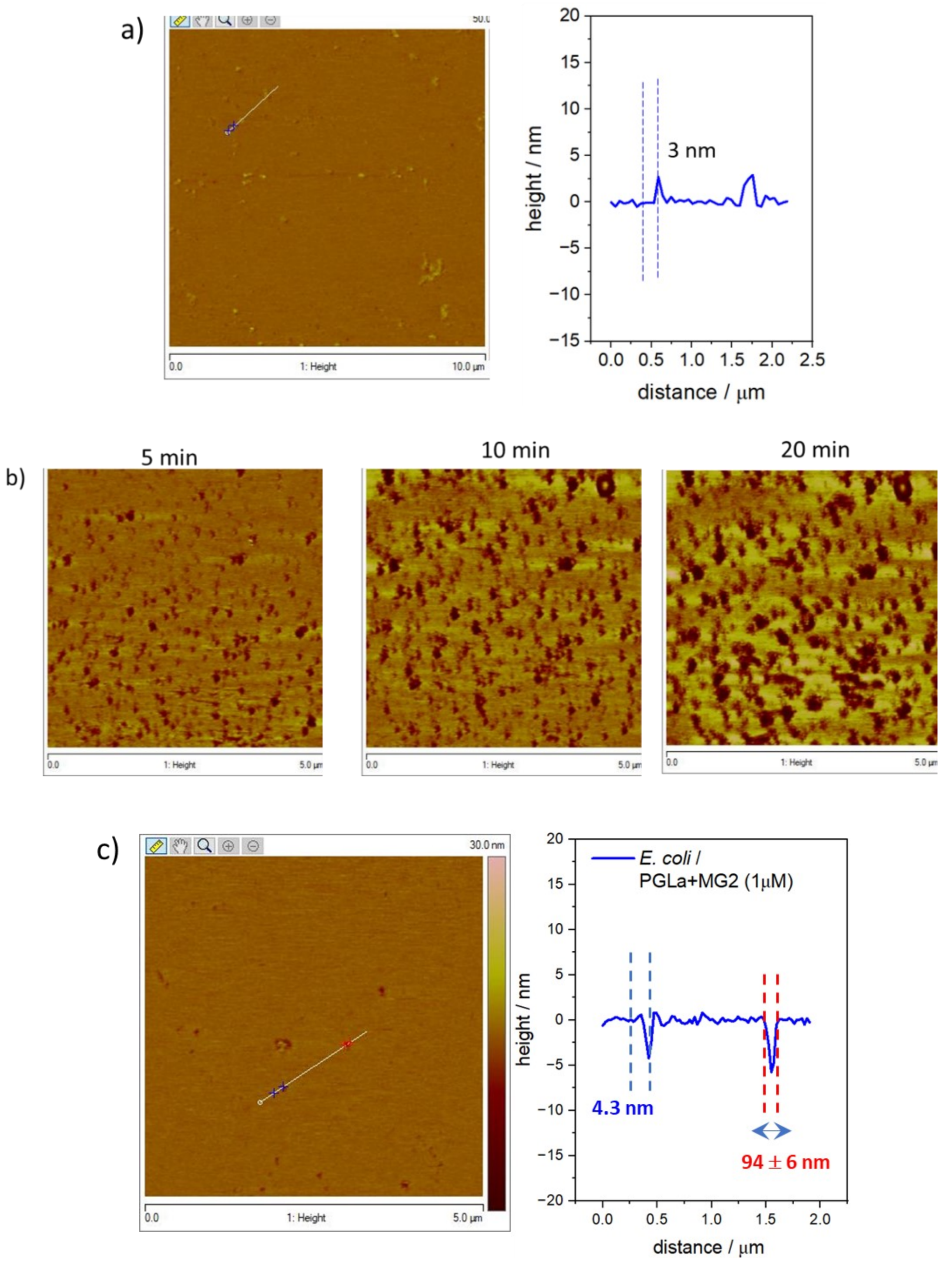

Figure S3: a) Topographic image (*left*) of *E. coli* bilayer and the corresponding line scan (*right*) taken after 1 h of incubation with 2 μM PGLa. b) Temporal evolution of topographic images of *E. coli* bilayer during the synergistic effect of equimolar mixture (1 μM Magainin 2 + 1 μM PGLa). c) Topographic image (*left*) of *E. coli* bilayer after 30 min incubation with equimolar mixture (0.5 μM PGLa + 0.5 μM Magainin 2) and the corresponding line profile analysis (*right*) quantifies the pore-formation.

1.6. FCS Measurements.

FCS measurements were performed on the labeled DOPE-ATTO532 and DOPE-ATTO655 to check the effect of AMPs or their mixture into the *E. coli* and mammalian model membrane MSLBs. Our FCS measurements were performed on a MicroTime 200 lifetime (PicoQuant GmbH, Berlin, Germany) using a water immersion objective (NA 1.2 UPlanSApo 60×1.2 CC1.48, Olympus). The detection unit comprises two single photon avalanche diodes from PicoQuant. A labeled lipid membrane marker DOPE-ATTO655 was excited with 640 nm LDH-P-C-640B (PicoQuant), and DOPE-ATTO532 was excited with a 532 nm PicoTA laser from Toptica (PicoQuant). To exclude scattered or reflected laser light, emitted fluorescence was collected through an HG670lp AHF/Chroma or HQ550lp AHF/Chroma band pass filter for 640 or 532 nm lasers, respectively. A 50 μm pinhole was used to eliminate photons from outside the confocal volume. Before FCS measurements, backscattered images of the substrate were taken using an OD3 density filter to ensure the optimal positioning of the focus to the center of the microcavity. Then, the bilayer position was determined by z-scanning until the point of maximal fluorescence intensity of DOPE-ATTO655 was found. At this point, the fluctuating fluorescence intensity of the labeled lipid marker or DOPE-ATTO532 was measured for 30 s per cavity with data averaged across of 10 to 20 cavities. To assess the diffusion time (ms) and the emitted photons were analyzed by a time-correlated single photon counting system (PicoHarp 300 from PicoQuant). The fluorescence fluctuations obtained are then correlated with a normalized autocorrelation function (eq 1)

$$G(\tau) = \frac{\langle \delta F(t)\delta F(t+\tau)\rangle}{\langle F(t)\rangle^2} \qquad (1)$$

The autocorrelation curves obtained from the fluorescence fluctuations of DOPE-ATTO655 and DOPE-ATTO532 in bilayer were fitted to a 2-D model (eq 2) using the software SymphoTime (SPT64) version 2.4 (PicoQuant).

$$G(\tau) = \frac{1}{N}\left[1 + \left(\frac{\tau}{\tau_D}\right)^{\alpha}\right]^{-1} \qquad (2)$$

Here, α is the anomalous parameter, N is the number of molecules and τ_D is the diffusion time of the fluorescent marked molecules in the lipid membrane. The diffusion coefficient is related to the correlation time τ_D by the relation $D = \omega^2/4\tau_D$, where ω is the $1/e^2$ radius of the confocal volume,

that is, the waist of the exciting laser beam. ω was measured for each excitation wavelength using a reference solution of free dye for which the diffusion coefficient is known. The ω was determined by calibration using reference dyes; ATTO655 (AttoTEC, GmbH) for a 640 nm laser and ATTO532 for a 532 nm laser at 20°C in water.

$$G(\tau) = \frac{1}{N}\left[1 + \left(\frac{\tau}{\tau_D}\right)^{\alpha}\right]^{-1}\left[1 + \left(\frac{\tau}{\tau_D}\right)^{\alpha}\frac{1}{\kappa^2}\right]^{-1/2} \quad (3)$$

The lateral diffusion of the free ATTO655 and ATTO532, e.g., unbound dye in PBS buffer, were calculated by fitting the ACFs obtained in for 10 nM solution of each molecule to a 3D model (eq. 3). Equation 3 includes a κ term, which defines the shape of the confocal volume.

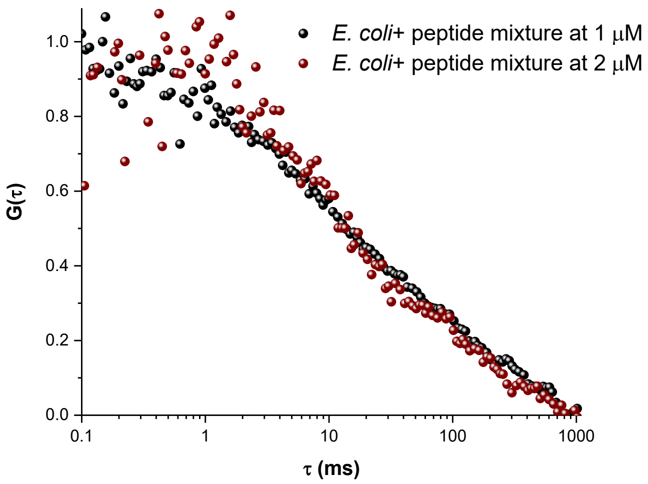

Figure S4: Representative autocorrelation functions (ACFs) obtained for labelled DOPE-ATTO655 incorporated into *E. coli* MSLB in presence of equimolar mixture of Magainin 2 and PGLa with 1 µM (black circles), 2 µM concentration (brown circles).

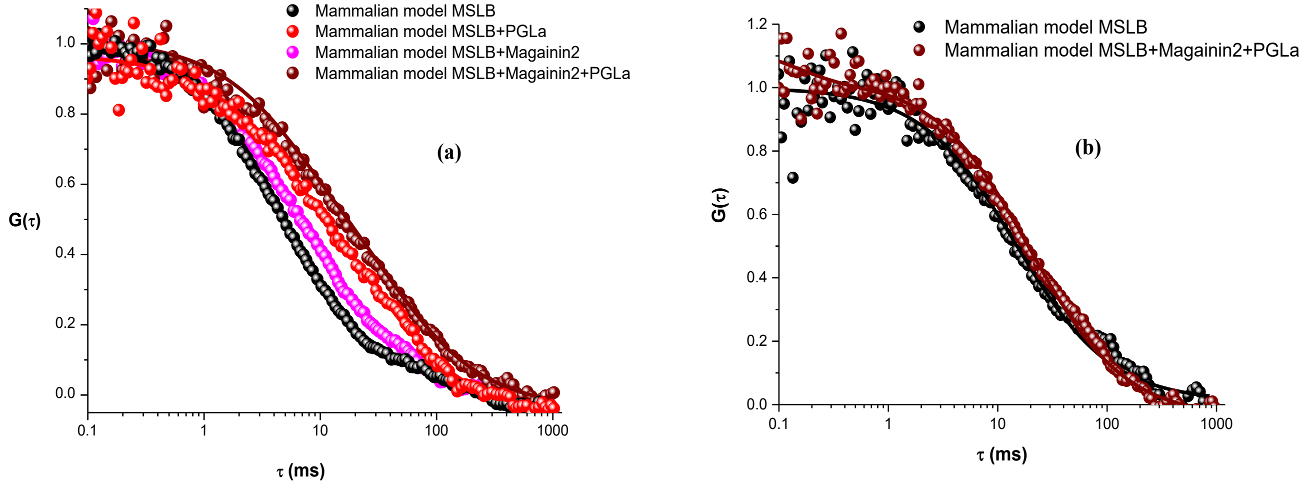

Figure S5: Representative autocorrelation functions (ACFs) obtained for labelled (a) DOPE-ATTO655 (in upper leaflet) incorporated into mammalian MSLB in absence of AMPs (black circles), in presence of PGLa (red circles), Magainin 2 (pink circles) and equimolar mixture of Magainin 2 and PGLa (brown circles) with 2 μM concentration in each case respectively, (b) ACFs obtained for labelled DOPE-ATTO532 (in lower leaflet) incorporated into mammalian MSLB in presence (brown circles) and absence of equimolar mixture of Magainin 2 and PGLa (black circle).

Table S1: Lateral diffusion coefficients of labelled DOPE-ATTO532 and DOPE-ATTO655 in *E. coli* MSLB system for the lower and upper leaflet respectively, obtained from FCS. The concentration of each individual AMP was 2 μM and the equimolar mixture of PGLa and Magainin 2 (total concentration 2 μM; 1 μM each)

System	*D* ($\mu m^2 s^{-1}$) DOPE-ATTO532	*D* ($\mu m^2 s^{-1}$) DOPE-ATTO655
E. coli	6.0 ± 0.64	7.90 ± 0.20
E. coli + Magainin 2	5.9 ± 0.80	6.10 ± 0.51
E. coli + PGLa	5.9 ± 0.52	2.30 ± 0.43 (90%), 0.10 ± 0.03 (10%)
E. coli + PGLa + Magainin 2	0.56 ± 0.26	1.84 ± 0.54 (80%), 0.10 ± 0.02 (20%)

Anomalous diffusion coefficient (α) is ~1 for all the composition.

Table S2: Lateral diffusion coefficients of labelled DOPE-ATTO532 and DOPE-ATTO655 in ternary MSLB system for the lower and upper leaflet respectively, obtained from FCS. The concentration of each individual AMP was 2 µM and the equimolar mixture of PGLa and Magainin 2 (total concentration 2 µM; 1 µM each)

System	D (µm^2 s^{-1}) DOPE-ATTO532	D (µm^2 s^{-1}) DOPE-ATTO655
DOPC: SM: Chol	3.10 ± 0.48	3.70 ± 0.58
DOPC: SM: Chol + Magainin 2	3.00 ± 0.50	3.30 ± 0.41
DOPC: SM: Chol + PGLa	3.10 ± 0.39	2.80 ± 0.37
DOPC: SM: Chol + PGLa + Magainin 2	3.10 ± 0.42	2.10 ± 0.43

Anomalous diffusion coefficient (α) is ~1 for all the composition.

1.7. Experimental section for Surface Enhanced Raman Spectroscopy (SERS) Measurements

The gold-microcavity-supported lipid bilayers in PBS at pH 7.4 were executed with Raman spectroscopy with a confocal microscope (Horiba, U.K.) and LabSpec software, LabSpec 5.45.09. A laser with 785 nm was used for excitation through a 600 µm pinhole equipped with a dispersion grating with 1200 grooves/mm. A 50× (air, NA:0.75) objective was used for both excitation and detection. The spectra of lipid bilayer were collected using 1 % laser, 0.1 mW (to avoid any damage to the bilayer) with an exposure time of 4 s and accumulation for 6 s. The instrument was calibrated using a Si (100) wafer calibrated to its standard peak at 520.6 cm^{-1} and the Rayleigh line before the measurement of the sample. The spectra of the powder form of the lipid were collected using a flat gold substrate with 10% laser power and 100 µm pinhole.

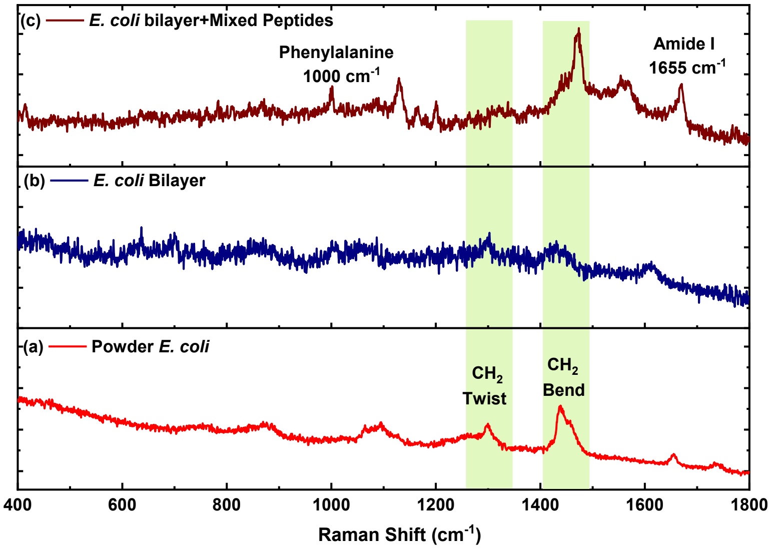

Figure S6: Surface Enhanced Raman Spectroscopy (SERS) of (b) *E. coli* bilayer and (c) *E. coli* bilayer in presence of mixed peptides on a 1 μm cavity array. In panel (a) classical Raman spectrum of *E. coli* in powder form (bulk) is shown.

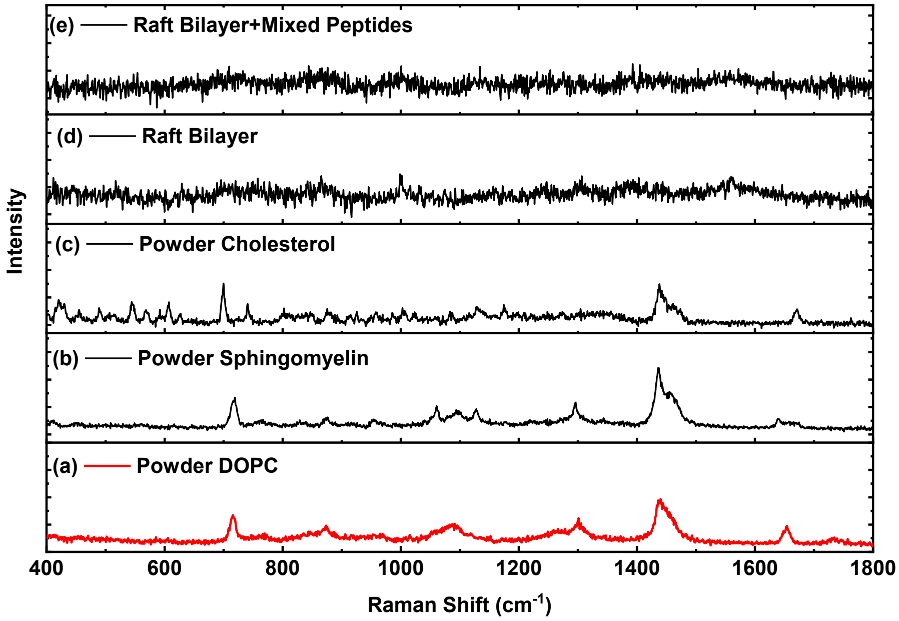

Figure S7: Surface Enhanced Raman Spectroscopy (SERS) of (d) raft bilayer and (e) raft bilayer in presence of mixed peptides on a 1 μm cavity array. In (a), (b) and (c) panel classical Raman spectra of Cholesterol, Sphingomyelin and DOPC in powder form is shown.

References:

(1) Gimenez, K. W.; Kho, A.V.; and Keyes, T. E. Nano-substructured plasmonic pore arrays: a robust, low cost route to reproducible hierarchical structures extended across macroscopic dimensions *Nanoscale Adv.*, **2020**, *2*, 4740–4756.

(2) Robinson, J.; Berselli, G. B.; Ryadnov, M. G.; and Keyes, T. E. Annexin V Drives Stabilization of Damaged Asymmetric Phospholipid Bilayers *Langmuir*, **2020**, *36*, 5454−5465.

(3) Maher, S.; Basit, H.; Forster, R. J.; and Keyes, T. E. Micron dimensioned cavity array supported lipid bilayers for the electrochemical investigation of ionophore activity *Bioelectrochemistry*, **2016**, *112*, 16–23.

(4) Basit, H.; Maher, S.; Forster, R. J.; and Keyes, T. E. Electrochemically Triggered Release of Reagent to the Proximal Leaflet of a Microcavity Supported Lipid Bilayer *Langmuir*, **2017**, *33*, 6691−6700.

(5) Sarangi, N. K.; Prabhakaran, A.; and Keyes, T. E. Multimodal Investigation into the Interaction of Quinacrine with Microcavity-Supported Lipid Bilayers *Langmuir*, **2022**, *38*, 6411–6424.

(6) Khan, M. S.; Dosoky, N. S.; and Williams, J. D. Engineering Lipid Bilayer Membranes for Protein Studies *Int. J. Mol. Sci.*, **2013**, *14*, 21561–21597.

(7) Khan, M. S.; Dosoky, N. S.; Berdiev, B. K. and Williams, J. D. Electrochemical impedance spectroscopy for black lipid membranes fused with channel protein supported on solid-state nanopore *Eur Biophys J*, **2016**, *45*, 843–852.
